# Emergency Department Use Among Adults Receiving Dialysis

**DOI:** 10.1001/jamanetworkopen.2024.13754

**Published:** 2024-05-29

**Authors:** Paul E. Ronksley, Tayler D. Scory, Andrew D. McRae, Jennifer M. MacRae, Braden J. Manns, Eddy Lang, Maoliosa Donald, Brenda R. Hemmelgarn, Meghan J. Elliott

**Affiliations:** 1 Department of Community Health Sciences, University of Calgary, Calgary, Alberta, Canada; 2 Department of Medicine, University of Calgary, Calgary, Alberta, Canada; 3 Department of Emergency Medicine, University of Calgary, Calgary, Alberta, Canada; 4 Department of Cardiac Sciences, University of Calgary, Calgary, Alberta, Canada; 5 Faculty of Medicine and Dentistry, University of Alberta, Edmonton, Alberta, Canada

## Abstract

**Importance:**

People with kidney failure receiving maintenance dialysis visit the emergency department (ED) 3 times per year on average, which is 3- to 8-fold more often than the general population. Little is known about the factors that contribute to potentially preventable ED use in this population.

**Objective:**

To identify the clinical and sociodemographic factors associated with potentially preventable ED use among patients receiving maintenance dialysis.

**Design, Setting, and Participants:**

This cohort study used linked administrative health data within the Alberta Kidney Disease Network to identify adults aged 18 years or older receiving maintenance dialysis (ie, hemodialysis or peritoneal dialysis) between April 1, 2010, and March 31, 2019. Patients who had been receiving dialysis for more than 90 days were followed up from cohort entry (defined as dialysis start date plus 90 days) until death, outmigration from the province, receipt of a kidney transplant, or end of study follow-up. The Andersen behavioral model of health services was used as a conceptual framework to identify variables related to health care need, predisposing factors, and enabling factors. Data were analyzed in March 2024.

**Main Outcomes and Measures:**

Rates of all-cause ED encounters and potentially preventable ED use associated with 4 kidney disease–specific ambulatory care–sensitive conditions (hyperkalemia, heart failure, volume overload, and malignant hypertension) were calculated. Multivariable negative binomial regression models were used to examine the association between clinical and sociodemographic factors and rates of potentially preventable ED use.

**Results:**

The cohort included 4925 adults (mean [SD] age, 60.8 [15.5] years; 3071 males [62.4%]) with kidney failure receiving maintenance hemodialysis (3183 patients) or peritoneal dialysis (1742 patients) who were followed up for a mean (SD) of 2.5 (2.0) years. In all, 3877 patients had 34 029 all-cause ED encounters (3100 [95% CI, 2996-3206] encounters per 1000 person-years). Of these, 755 patients (19.5%) had 1351 potentially preventable ED encounters (114 [95% CI, 105-124] encounters per 1000 person-years). Compared with patients with a nonpreventable ED encounter, patients with a potentially preventable ED encounter were more likely to be in the lowest income quintile (38.8% vs 30.9%; *P* < .001); to experience heart failure (46.8% vs 39.9%; *P* = .001), depression (36.6% vs 32.5%; *P* = .03), and chronic pain (60.1% vs 54.9%; *P* = .01); and to have a longer duration of dialysis (3.6 vs 2.6 years; *P* < .001). In multivariable regression analyses, potentially preventable ED use was higher for younger adults (incidence rate ratio [IRR], 1.69 [95% CI, 1.33-2.15] for those aged 18 to 44 years) and patients with chronic pain (IRR, 1.35 [95% CI, 1.14-1.61]), greater material deprivation (IRR, 1.57 [95% CI, 1.16-2.12]), a history of hyperkalemia (IRR, 1.31 [95% CI, 1.09-1.58]), and historically high ED use (ie, ≥3 ED encounters in the prior year; IRR, 1.46 [95% CI, 1.23-1.73).

**Conclusions and Relevance:**

In this study of adults receiving maintenance dialysis in Alberta, Canada, among those with ED use, 1 in 5 had a potentially preventable ED encounter; reasons for such encounters were associated with both psychosocial and medical factors. The findings underscore the need for strategies that address social determinants of health to avert potentially preventable ED use in this population.

## Key Points

**Question:**

What medical and sociodemographic factors are associated with potentially preventable emergency department (ED) use among people receiving maintenance dialysis?

**Findings:**

In this cohort study of 4925 adults receiving maintenance dialysis in Alberta, Canada, rates of potentially preventable ED use were higher for individuals aged 44 years or younger and those with chronic pain, greater material deprivation, previous hyperkalemia, and historically high ED use.

**Meaning:**

Findings of this study suggest that strategies to mitigate potentially preventable ED use in patients receiving maintenance dialysis should consider individuals’ psychosocial and medical factors.

## Teaser

This cohort study assesses factors associated with potentially preventable emergency department use by adults receiving maintenance dialysis in Canada.

## Introduction

The considerable increase in emergency department (ED) use across North America over the past decade has strained the capacity of health systems to respond to urgent health concerns in a timely and effective way.^1,2^ Many efforts to improve the appropriateness of ED use and prevent the need for emergency care focus on frequent ED users, who reflect a minority of all ED users but a disproportionately high number of ED presentations.^3^ Observational reports suggest people with kidney failure receiving dialysis are frequent ED users, with a mean of 3 ED encounters per year and reported rates of ED use up to 8 times greater than the general population.^4,5^ In addition to patients with kidney failure being older, more frail, and having more comorbid conditions than the general public, their reliance on dialysis as life-sustaining therapy both contributes to and complicates the high ED use observed in this population.^6^

People with kidney failure receive emergency care for a variety of health issues that occur both with and as a result of their underlying kidney disease.^7^ Studies have noted temporal trends in increased acute care use during specific periods, such as after long interdialytic intervals and shortly before and after initiating dialysis.^5,8,9,10,11^ However, the medical reasons prompting ED visits are broadly distributed across many diagnoses that can be but are not necessarily related to kidney failure or the need for dialysis.^12^ Previous research by our group has found that 6% of all ED encounters among people with chronic kidney disease were for conditions commonly attributed to advanced kidney disease that are potentially preventable through early identification and intervention (eg, hyperkalemia, volume overload).^4^ The higher rates of potentially preventable ED use and hospitalizations observed among people receiving dialysis compared with those with earlier stages of kidney disease^4,13^ underscore a need to understand the individual and contextual drivers in this complex population.

Frequent ED use has been associated with socioeconomic disadvantage, low primary care attachment, greater use of other health care services, and chronic disease and mental health burden among ED all comers.^14,15,16,17^ However, the factors associated with potentially preventable ED encounters among people receiving dialysis are poorly understood yet necessary to identify and enable the design of interventions that improve appropriateness of acute care use, system efficiencies, and patient outcomes and experiences. In this population-based study, we used linked administrative health data to determine rates and characteristics of potentially preventable ED encounters among a cohort of patients with kidney failure receiving maintenance dialysis.

## Methods

### Study Design and Population

We conducted a retrospective cohort study using linked laboratory and administrative health data within the Alberta Kidney Disease Network database.^18^ This study adheres to the Strengthening the Reporting of Observational Studies in Epidemiology (STROBE) reporting guideline and the Reporting of studies Conducted using Observational Routinely-collected health Data (RECORD) statement.^19^ The study received ethics approval and a waiver of patient informed consent from the University of Calgary Conjoint Health Research Ethics Board because the large sample size and geographic range would make obtaining consent unfeasible.

The study population included all Alberta residents aged 18 years and older who were receiving maintenance dialysis (ie, hemodialysis or peritoneal dialysis) between April 1, 2010, and March 31, 2019. Receipt of dialysis was identified within the provincial dialysis registries (Alberta Kidney Care–North and –South) for more than 90 days to establish maintenance dialysis status and to prevent misclassification of people receiving short-term dialysis for acute kidney injury.^20^ All patients were followed up from cohort entry (defined as dialysis start date plus 90 days) until death, outmigration from the province, receipt of a kidney transplant, or end of study follow-up. We excluded patients with a prior transplant and those who died or received a kidney transplant between the start of dialysis and cohort entry date.

### Outcomes

We linked the cohort to provincial administrative data sources via each individual’s unique provincial health care number. The National Ambulatory Care Reporting System was used to count the number of all-cause ED encounters from cohort entry until death, outmigration from the province, receipt of a kidney transplant, or end of study follow-up. Encounter-level information was extracted, including the top 10 most frequent ED diagnoses and the proportion of ED visits resulting in hospital admission or death. Multiple encounters on the same day were reported as 1 event, with information being reported from the first event.

We defined potentially preventable ED use as encounters for kidney disease–specific ambulatory care–sensitive conditions (ACSCs). We defined ACSCs as health conditions for which adequate outpatient care can prevent the need for acute care presentation and are recognized internationally as a measure of the adequacy of ambulatory and primary health care performance.^21,22^ Using established coding algorithms based on *International Classification of Diseases, Ninth Revision, Clinical Modification* (*ICD-9-CM)* and *International Statistical Classification of Diseases and Related Health Problems, Tenth Revision, Canada (ICD-10-CA)*, we focused on 4 previously defined kidney disease–specific ACSCs (hyperkalemia, malignant hypertension, heart failure, and volume overload) listed as the ED diagnosis code responsible for the ED encounter (eTable 1 in Supplement 1).^23,24^ Heart failure and volume overload are distinguished in these coding algorithms by the latter comprising extracellular fluid volume expansion that is not attributed directly to poor cardiac function.

### Covariates

Included patients were linked to additional administrative data sources obtained from the provincial health ministry to capture sociodemographic data, clinical characteristics, and measures of prior health care use. We used the Andersen behavioral model of health services use as a conceptual framework to identify variables related to health care need, predisposing factors, and enabling factors, where available, from our data sources.^25^ Patient-level characteristics were measured at cohort entry date except for laboratory values, which were measured at the date of the first ED encounter. Sociodemographic characteristics included age, sex, urban or rural status (based on the Statistics Canada definition of rural residence as a population <1000 or a population density <400/km^2^), median neighborhood income quintile, and Pampalon material and social deprivation indices, which are area-level composite measures derived from census data. The material index reflects deprivation of wealth, goods, and conveniences, while the social index reflects deprivation of relationships in the family, workplace, and community.^26^ These indices are categorized into quintiles, with quintile 1 indicating the lowest and quintile 5 the greatest material and social deprivation.

We reported on comorbidities including previous acute myocardial infarction, atrial fibrillation, cancer, chronic pain, chronic pulmonary disease, severe constipation, depression, dementia, diabetes, heart failure, hypertension, hypothyroidism, peripheral vascular disease, and stroke. These were defined using validated algorithms of *ICD-9-CM* and *ICD-10-CA* codes (eTable 1 in Supplement 1).^27^ Dialysis-related characteristics, such as initial modality, duration (in years), and modality switch (ie, switch from hemodialysis to peritoneal dialysis or vice versa at any time during follow-up), were identified within the provincial dialysis registries. Outpatient laboratory tests were also reported using the most recent measurement within the 6 months before the date of the first ED encounter. These included hemoglobin A_1c_, serum potassium, and serum sodium levels. Cutoff values for laboratory parameters were defined according to the Medical Council of Canada.^28^ Health care resource use in the year prior to cohort entry was also measured and included the number of primary care visits, primary care attachment (ie, usual provider of care index),^29^ number of cardiologist visits, number of prior ED visits, number of hospitalizations, and characteristics of the hospitalizations (ie, cumulative length of stay, intensive care unit admission, or long-term care placement at discharge).

### Statistical Analysis

Patient characteristics were summarized using counts for categorical and dichotomous variables and means and SDs for continuous variables. All variables of interest were stratified by rate of ED use, which was operationalized as 0 visits per person-year (ie, no ED encounters during follow-up), less than 1 visit per person-year, 1 to less than 3 visits per person-year, and 3 or more ED visits per person-year. Crude rates of all-cause ED encounters by year of cohort entry were also calculated to explore trends in ED use over time. In a separate analysis, patient characteristics were stratified by the presence or absence of at least 1 ACSC ED presentation during the follow-up period. All statistical comparisons for potential differences in patient clinical and sociodemographic characteristics were performed using χ^2^ tests, *t* tests, and analysis of variance, as appropriate. When covariate data were missing, a category was included for missing data.

Crude rates of all-cause and kidney-specific ACSC ED use (per 1000 person-years) were calculated using generalized linear models with a negative binomial distribution and log link. To identify patient and clinical factors associated with rates of potentially preventable ED encounters, multivariable negative binomial regression models were created initially among those with at least 1 ED encounter. In sensitivity analyses, models were then rerun among the entire cohort (including those with no ED encounters) to assess the robustness of study findings. Factors that were statistically significant in univariable models were included in a multivariable model. A parsimonious model was then developed using backward elimination, with the Akaike information criterion and likelihood ratio test used to determine model goodness of fit.^30^ All variables in the final adjusted model were reported as incident rate ratios (IRRs). For all statistical tests, *P* < .05 was considered statistically significant. Data were analyzed in March 2024 using SAS, version 9.4 (SAS Institute Inc).

## Results

**Figure 1.**
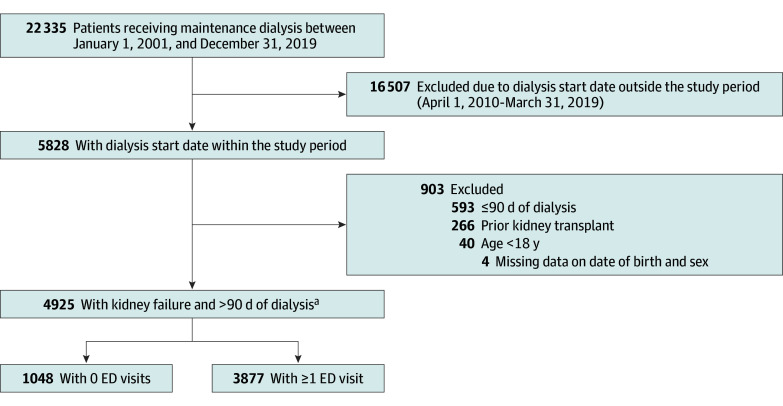
Flowchart of Study Cohort ED indicates emergency department. ^a^Time on dialysis was calculated during the study period only.

We identified 4925 adults (mean [SD] age, 60.8 [15.5] years; 3071 males [62.4%] and 1854 females [37.6%]) with kidney failure between April 1, 2010, and March 31, 2019 (Figure 1). Of the total cohort, 3877 patients (78.7%) had at least 1 ED encounter. There were 34 029 unique ED encounters among these 3877 patients over a mean (SD) follow-up of 2.5 (2.0) years. The rate of all-cause ED encounters was 3100 (95% CI, 2996-3206) per 1000 person-years.

Nearly one-third of patients (28.3% and 29.5%) scored in the highest quintiles (ie, most deprived) on the Pampalon social and material deprivation indices, respectively (Table 1). More than half (64.6%) of the cohort was receiving maintenance hemodialysis at study entry, and the mean (SD) duration of dialysis was 2.4 (2.0) years. The most common comorbidities among the cohort were hypertension (90.7%), diabetes (61.0%), chronic pain (53.4%), and heart failure (39.0%). Approximately one-third (37.8%) died during the study.

**Table 1.** Demographic and Clinical Characteristics of Adults Receiving Dialysis Between April 1, 2010, and March 31, 2019, Overall and Stratified by Rate of All-Cause ED Encounters^a^

Characteristic	Overall, No. (%)	All-cause ED encounters per person-year, No. (%)				*P* value^b^
		0	<1	1 to <3	≥3	
Total patients	4925	1048	773	1498	1606	NA
Demographic characteristics						
Age, mean (SD), y	60.8 (15.5)	59.6 (15.6)	60.5 (15.1)	61.9 (15.4)	60.8 (15.5)	.002
Age group, y						
18 to 44	805 (16.3)	192 (18.3)	128 (16.6)	217 (14.5)	268 (16.7)	.01
45 to 64	2011 (40.8)	435 (41.5)	341 (44.1)	590 (39.4)	645 (40.2)	
≥ 65	2109 (42.8)	421 (40.2)	304 (39.3)	691 (46.1)	693 (43.2)	
Sex						
Male	3071 (62.4)	700 (66.8)	496 (64.2)	926 (61.8)	949 (59.1)	<.001
Female	1854 (37.6)	348 (33.2)	277 (35.8)	572 (38.2)	657 (40.9)	
Residence^c^						
Urban	3986 (80.9)	887 (84.6)	684 (88.5)	1270 (84.8)	1145 (71.3)	<.001
Rural	921 (18.7)	154 (14.7)	88 (11.4)	224 (15.0)	455 (28.3)	
Missing data	18 (0.4)	7 (0.7)	NR	NR	6 (0.4)	
Income quintile, before tax						
1 (lowest)	1558 (31.6)	301 (28.7)	198 (25.6)	523 (34.9)	536 (33.4)	<.001
2	1135 (23.0)	221 (21.1)	196 (25.4)	333 (22.2)	385 (24.0)	
3	840 (17.1)	181 (17.3)	150 (19.4)	241 (16.1)	268 (16.7)	
4	743 (15.1)	164 (15.6)	136 (17.6)	216 (14.4)	227 (14.1)	
5 (highest)	630 (12.8)	174 (16.6)	92 (11.9)	181 (12.1)	183 (11.4)	
Missing data	19 (0.4)	7 (0.7)	NR	NR	7 (0.4)	
Pampalon material deprivation index						
1 (least deprived)	569 (11.6)	156 (14.9)	100 (12.9)	168 (11.2)	145 (9.0)	<.001
2	705 (14.3)	167 (15.9)	123 (15.9)	220 (14.7)	195 (12.1)	
3	865 (17.6)	186 (17.7)	148 (19.1)	265 (17.7)	266 (16.6)	
4	986 (20.0)	214 (20.4)	161 (20.8)	297 (19.8)	314 (19.6)	
5 (most deprived)	1455 (29.5)	271 (25.9)	201 (26.0)	448 (29.9)	535 (33.3)	
Missing data	345 (7.0)	54 (5.2)	40 (5.2)	100 (6.7)	151 (9.4)	
Pampalon social deprivation index						
1 (least deprived)	724 (14.7)	194 (18.5)	134 (17.3)	199 (13.3)	197 (12.3)	<.001
2	594 (12.1)	132 (12.6)	114 (14.7)	188 (12.6)	160 (10.0)	
3	839 (17.0)	181 (17.3)	148 (19.1)	233 (15.6)	277 (17.2)	
4	1030 (20.9)	194 (18.5)	148 (19.1)	344 (23.0)	344 (21.4)	
5 (most deprived)	1393 (28.3)	293 (28)	189 (24.5)	434 (29.0)	477 (29.7)	
Missing data	345 (7.0)	54 (5.2)	40 (5.2)	100 (6.7)	151 (9.4)	
Comorbidities						
Acute myocardial infarction	525 (10.7)	97 (9.3)	56 (7.2)	149 (9.9)	223 (13.9)	<.001
Atrial fibrillation	773 (15.7)	152 (14.5)	78 (10.1)	241 (16.1)	302 (18.8)	<.001
Cancer	427 (8.7)	91 (8.7)	47 (6.1)	120 (8.0)	169 (10.5)	.002
Chronic pain	2630 (53.4)	461 (44.0)	314 (40.6)	822 (54.9)	1033 (64.3)	<.001
Chronic pulmonary disease	1403 (28.5)	213 (20.3)	136 (17.6)	432 (28.8)	622 (38.7)	<.001
Constipation, severe	568 (11.5)	79 (7.5)	54 (7.0)	169 (11.3)	266 (16.6)	<.001
Depression	1576 (32.0)	285 (27.2)	168 (21.7)	480 (32.0)	643 (40.0)	<.001
Dementia	248 (5.0)	51 (4.9)	32 (4.1)	79 (5.3)	86 (5.4)	.60
Diabetes	3004 (61.0)	550 (52.5)	401 (51.9)	927 (61.9)	1126 (70.1)	<.001
Heart failure	1920 (39.0)	322 (30.7)	247 (32.0)	586 (39.1)	765 (47.6)	<.001
Hypertension	4466 (90.7)	914 (87.2)	680 (88.0)	1379 (92.1)	1493 (93.0)	<.001
Hypothyroidism	711 (14.4)	132 (12.6)	94 (12.2)	223 (14.9)	262 (16.3)	.01
Peripheral vascular disease	594 (12.1)	97 (9.3)	85 (11.0)	187 (12.5)	225 (14.0)	.002
Stroke	1053 (21.4)	160 (15.3)	141 (18.2)	343 (22.9)	409 (25.5)	<.001
Dialysis characteristics						
Initial dialysis modality						
Hemodialysis	3183 (64.6)	684 (65.3)	476 (61.6)	939 (62.7)	1084 (67.5)	.009
Peritoneal dialysis	1742 (35.4)	364 (34.7)	297 (38.4)	559 (37.3)	522 (32.5)	
Other characteristics						
Follow-up, mean (SD), y	2.5 (2.0)	1.1 (1.3)	3.6 (1.8)	3.2 (2.0)	2.1 (1.8)	<.001
Death during follow-up						
No	3064 (62.2)	874 (83.4)	592 (76.6)	878 (58.6)	720 (44.8)	<.001
Yes	1861 (37.8)	174 (16.6)	181 (23.4)	620 (41.4)	886 (55.2)	
Potassium level^d^						
Low (<3.5 mEq/L)	307 (6.2)	NA	66 (8.5)	118 (7.9)	123 (7.7)	.93
Normal (3.5 to <5.0 mEq/L)	2562 (52.0)	NA	501 (64.8)	998 (66.6)	1063 (66.2)	
High (≥5.0 mEq/L)	985 (20.0)	NA	198 (25.6)	375 (25.0)	412 (25.7)	
No test	1071 (21.7)	1048 (100)	8 (1.0)	7 (0.5)	8 (0.5)	
Sodium level^d^						
Low (<135 mEq/L)	1562 (31.7)	NA	316 (40.9)	609 (40.7)	637 (39.7)	.96
Normal (135 to <145 mEq/L)	2260 (45.9)	NA	442 (57.2)	870 (58.1)	948 (59.0)	
High (≥145 mEq/L)	35 (0.7)	NA	7 (0.9)	13 (0.9)	15 (0.9)	
No test	1068 (21.7)	1048 (100)	8 (1.0)	6 (0.4)	6 (0.4)	
Hemoglobin A_1c_ level^d^						
Normal (<6.0%)	962 (19.5)	NA	208 (26.9)	340 (22.7)	414 (25.8)	.003
Prediabetic (6% to <6.5%)	318 (6.5)	NA	49 (6.3)	126 (8.4)	143 (8.9)	
Diabetic (≥6.5%)	1411 (28.6)	NA	220 (28.5)	538 (35.9)	653 (40.7)	
No test	2234 (45.4)	1048 (100)	296 (38.3)	494 (33.0)	396 (24.7)	

Abbreviations: ED, emergency department; NA, not applicable; NR, not reported.

SI conversion factors: To convert potassium to millimoles per liter, multiply by 1; to convert sodium to millimoles per liter, multiply by 1; to convert hemoglobin A_1c_ to proportion of total hemoglobin, multiply by 0.01.

^a^ Study characteristics except laboratory values were measured at cohort entry (90 days after initial dialysis date). Laboratory values were measured at first ED encounter. Cells with n <5 are masked for privacy and indicated by NR.

^b^ *P* values result from χ^2^ tests and analysis of variance for categorical and continuous variables, respectively. Patients with 0 ED encounters per person-year were included in the statistical testing.

^c^ Residence is based on the Statistics Canada definition of rural residence (population <1000 or a population density <400/km^2^).

^d^ Based on the most recent laboratory measurement preceding the date of the first ED encounter within a 6-month timeframe.

There were 1048 patients (21.3%) who had 0 ED encounters during the study period and thus a rate of 0 all-cause ED visits per year. There were 773 patients (15.7%) with less than 1 ED visit per year, 1498 (30.4%) with 1 to less than 3 ED visits per year, and 1606 (32.6%) with 3 or more ED visits per year. Throughout the study period, the rate of ED visits remained stable (approximately 3 visits per person-year) (Table 1). The proportion of patients who were female and those living in rural locations within the province increased as the rate of ED use increased. Furthermore, the proportions of patients in the highest material and social deprivation quintiles and lowest income quintile also increased as the rate of ED use increased (Table 1). The prevalence of all measured comorbidities increased with increasing rates of ED use; for example, chronic pain was present in 44.0% of patients with 0 ED visits per person-year and 64.3% of those with 3 or more ED visits per person-year (*P* < .001). Patients receiving maintenance dialysis who had the highest rates of ED use (≥3 ED encounters per year) were more likely to die during the study than those with low rates of ED use (<1 ED encounter per year) (55.2% vs 23.4%, respectively).

Of the 3877 patients with at least 1 ED encounter, 755 (19.5%) had 1 or more ACSC presentations and a total of 1351 unique ACSC encounters during the study follow-up period (adjusted rate of 114 (95% CI, 105-124) events per 1000 person-years) (Table 2). Among those with an ACSC presentation, the 2 most common types of ACSCs were hyperkalemia and heart failure, with rates of 46.3 (95% CI, 40.9-52.5) and 46.2 (95% CI, 41.2-51.8) per 1000 person-years, respectively. Patients receiving dialysis with at least 1 kidney disease–specific ACSC were more likely to be materially and socially disadvantaged, have a higher comorbidity burden (most notably for the prevalence of heart failure, chronic pain, and depression), and have a longer duration of dialysis compared with those with no ACSC ED encounters (Table 2). They were also more likely to die during the study timeframe (41.9% for those without an ACSC vs 50.3% for those with an ACSC). Fifty-one percent (689 of 1351) of ED encounters for an ACSC resulted in a hospital admission compared with 28.8% (9396 of 32 678) for a non-ACSC–related ED encounter (*P* < .001). Hospital length of stay was slightly shorter for ACSC-related ED encounters resulting in admission than for non-ACSC–related admissions (median [IQR], (5 [3-9] days vs 7 [4-15] days). Differences in the top 10 diagnoses most responsible for the ED encounter (patient- and encounter-level analyses) for the first ED encounter and any ED encounter stratified by ACSC status are provided in eTables 2 and 3 in Supplement 1.

**Table 2.** Demographic Characteristics of Adults Receiving Maintenance Dialysis Between April 1, 2010, and March 31, 2019, Overall and Stratified by Presence of ACSC ED Encounter^a^

Characteristic	No. (%)				*P* value^b^
	Overall	No ED encounters	Non-ACSC ED encounter	ACSC ED encounter	
Total adults	4925	1048	3122	755	NA
Demographic characteristics					
Age, mean (SD), y	60.8 (15.5)	59.6 (15.6)	61.4 (15.3)	60.3 (15.7)	.22
Age group, y					
18 to 44	805 (16.3)	192 (18.3)	480 (15.4)	133 (17.6)	.31
45 to 64	2011 (40.8)	435 (41.5)	1274 (40.8)	302 (40.0)	
≥ 65	2109 (42.8)	421 (40.2)	1368 (43.8)	320 (42.4)	
Sex					
Male	3071 (62.4)	700 (66.8)	1908 (61.1)	463 (61.3)	.92
Female	1854 (37.6)	348 (33.2)	1214 (38.9)	292 (38.7)	
Residence^c^					
Urban	3986 (80.9)	887 (84.6)	2508 (80.3)	591 (78.3)	.20
Rural	921 (18.7)	154 (14.7)	605 (19.4)	162 (21.5)	
Missing data	18 (0.4)	7 (0.7)	9 (0.3)	NR	
Income quintile, before tax					
1 (lowest)	1558 (31.6)	301 (28.7)	964 (30.9)	293 (38.8)	<.001
2	1135 (23.0)	221 (21.1)	734 (23.5)	180 (23.8)	
3	840 (17.1)	181 (17.3)	548 (17.6)	111 (14.7)	
4	743 (15.1)	164 (15.6)	480 (15.4)	99 (13.1)	
5 (highest)	630 (12.8)	174 (16.6)	386 (12.4)	70 (9.3)	
Missing data	19 (0.4)	7 (0.7)	10 (0.3)	NR	
Pampalon material deprivation index					
1 (least deprived)	569 (11.6)	156 (14.9)	347 (11.1)	66 (8.7)	.002
2	705 (14.3)	167 (15.9)	431 (13.8)	107 (14.2)	
3	865 (17.6)	186 (17.7)	552 (17.7)	127 (16.8)	
4	986 (20.0)	214 (20.4)	645 (20.7)	127 (16.8)	
5 (most deprived)	1455 (29.5)	271 (25.9)	912 (29.2)	272 (36.0)	
Missing data	345 (7.0)	54 (5.2)	235 (7.5)	56 (7.4)	
Pampalon social deprivation index					
1 (least deprived)	724 (14.7)	194 (18.5)	431 (13.8)	99 (13.1)	.12
2	594 (12.1)	132 (12.6)	388 (12.4)	74 (9.8)	
3	839 (17.0)	181 (17.3)	537 (17.2)	121 (16.0)	
4	1030 (20.9)	194 (18.5)	667 (21.4)	169 (22.4)	
5 (most deprived)	1393 (28.3)	293 (28.0)	864 (27.7)	236 (31.3)	
Missing data	345 (7.0)	54 (5.2)	235 (7.5)	56 (7.4)	
Comorbidities					
Acute myocardial infarction	525 (10.7)	97 (9.3)	321 (10.3)	107 (14.2)	.002
Atrial fibrillation	773 (15.7)	152 (14.5)	497 (15.9)	124 (16.4)	.73
Cancer	427 (8.7)	91 (8.7)	296 (9.5)	40 (5.3)	<.001
Chronic pain	2630 (53.4)	461 (44.0)	1715 (54.9)	454 (60.1)	.01
Chronic pulmonary disease	1403 (28.5)	213 (20.3)	933 (29.9)	257 (34.0)	.03
Constipation, severe	568 (11.5)	79 (7.5)	371 (11.9)	118 (15.6)	.005
Depression	1576 (32.0)	285 (27.2)	1015 (32.5)	276 (36.6)	.03
Dementia	248 (5.0)	51 (4.9)	159 (5.1)	38 (5.0)	.95
Diabetes mellitus	3004 (61.0)	550 (52.5)	1923 (61.6)	531 (70.3)	<.001
Heart failure	1920 (39.0)	322 (30.7)	1245 (39.9)	353 (46.8)	.001
Hypertension	4466 (90.7)	914 (87.2)	2847 (91.2)	705 (93.4)	.05
Hypothyroidism	711 (14.4)	132 (12.6)	470 (15.1)	109 (14.4)	.67
Peripheral vascular disease	594 (12.1)	97 (9.3)	399 (12.8)	98 (13.0)	.88
Stroke	1053 (21.4)	160 (15.3)	720 (23.1)	173 (22.9)	.93
Dialysis characteristics					
Initial dialysis modality					
Hemodialysis	3183 (64.6)	684 (65.3)	2002 (64.1)	497 (65.8)	.38
Peritoneal dialysis	1742 (35.4)	364 (34.7)	1120 (35.9)	258 (34.2)	
Modality switch^d^					
No	3756 (76.3)	890 (84.9)	2332 (74.7)	534 (70.7)	.03
Yes	1169 (23.7)	158 (15.1)	790 (25.3)	221 (29.3)	
Duration of dialysis, mean (SD), y	2.4 (2.0)	1.1 (1.3)	2.6 (1.9)	3.6 (2.0)	<.001
Other characteristics					
Follow-up, mean (SD), y	2.5 (2.0)	1.1 (1.3)	2.6 (1.9)	3.6 (2.0)	<.001
Death					
No	3064 (62.2)	874 (83.4)	1815 (58.1)	375 (49.7)	<.001
Yes	1861 (37.8)	174 (16.6)	1307 (41.9)	380 (50.3)	
Potassium level^e^					
Low (<3.5 mEq/L)	307 (6.2)	NA	264 (8.5)	43 (5.7)	<.001
Normal (3.5 to <5.0 mEq/L)	2562 (52.0)	NA	2094 (67.1)	468 (62.0)	
High (≥5.0 mEq/L)	985 (20.0)	NA	746 (23.9)	239 (31.7)	
No test	1071 (21.7)	1048 (100)	18 (0.6)	5 (0.7)	
Sodium level^e^					
Low (<135 mEq/L)	1562 (31.7)	NA	1264 (40.5)	298 (39.5)	.78
Normal (135 to <145 mEq/L)	2260 (45.9)	NA	1814 (58.1)	446 (59.1)	
High (≥145 mEq/L)	35 (0.7)	NA	27 (0.9)	8 (1.1)	
No test	1068 (21.7)	1048 (100)	17 (0.5)	NR	
Hemoglobin A_1c_ level^e^					
Normal (<6.0%)	962 (19.5)	NA	787 (25.2)	175 (23.2)	.01
Prediabetic (6.0% to <6.5%)	318 (6.5)	NA	256 (8.2)	62 (8.2)	
Diabetic (≥6.5%)	1411 (28.6)	NA	1085 (34.8)	326 (43.2)	
No test	2234 (45.4)	1048 (100)	994 (31.8)	192 (25.4)	

Abbreviations: ACSC, ambulatory care–sensitive condition; ED, emergency department; NA, not applicable; NR, not reported.

SI conversion factors: To convert potassium to millimoles per liter, multiply by 1; to convert hemoglobin A_1c_ to proportion of total hemoglobin, multiply by 0.01.

^a^ Study characteristics except laboratory values were measured at cohort entry (90 days after initial dialysis date). Laboratory values were measured at first ED encounter. Cells with n <5 are masked for privacy and indicated by NR.

^b^ *P* values result from χ^2^ tests and pooled *t* tests for categorical and continuous variables, respectively. Patients who did not have an ED encounter were not included in the comparison.

^c^ Residence is based on the Statistics Canada definition of rural residence (population <1000 or a population density <400/km^2^).

^d^ Patients who experienced at least 1 modality switch at any time during follow-up.

^e^ Based on the most recent laboratory measurement preceding the date of the first ED encounter within a 6-month timeframe.

**Figure 2.**
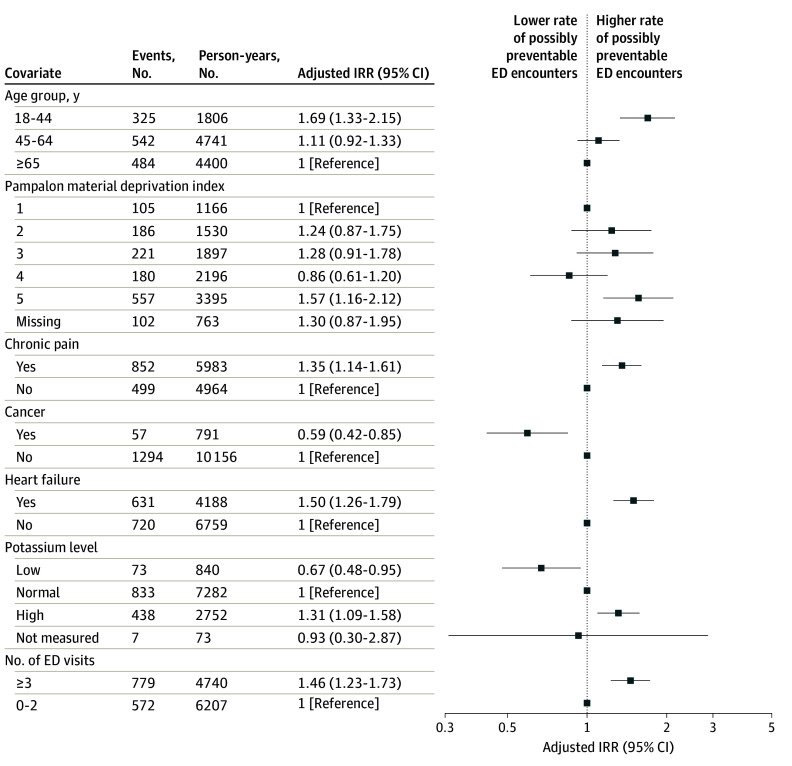
Factors Associated With Potentially Preventable Emergency Department (ED) Encounters The results are based on a multivariable regression model that was adjusted for the covariates listed. Pampalon deprivation index ranked from 1 (lowest) to 5 (highest). Potassium levels categorized as low (<3.5 mEq/L [to convert to millimoles per liter, multiply by 1]), normal (3.5 to <5.0 mEq/L), or high (≥5.0 mEq/L). IRR indicates incident rate ratio.

Multivariable regression modeling showed that rates of ACSC-related ED encounters were significantly higher for younger patients (IRR, 1.69 [95% CI, 1.33-2.15] for those 18-44 years vs ≥65 years) and those with chronic pain (IRR, 1.35 [95% CI, 1.14-1.61] vs those without chronic pain) and heart failure (IRR, 1.50 [95% CI, 1.26-1.79] vs those without heart failure). Patients with a history of cancer within 5 years prior to cohort entry had lower rates of ACSC-related ED use (IRR, 0.59 [95% CI, 0.42-0.85] compared with patients without a cancer diagnosis). Patients with the highest level of material deprivation also had higher rates of ACSCs (IRR, 1.57 [95% CI, 1.16-2.12] vs those with the lowest level of material deprivation). Finally, patients with a history of hyperkalemia (at the measurement closest to the ED encounter within the previous 6 months) and historically high ED use (defined as 3 or more ED encounters in the prior year) had higher rates of ACSC presentation (Figure 2; eTable 4 in Supplement 1). In sensitivity analyses, results were similar for regression models that included the subset of patients with no ED encounters (eFigure and eTable 5 in Supplement 1).

## Discussion

In this large, population-based cohort, we found that patients receiving maintenance dialysis experienced approximately 3 all-cause ED encounters per year. While rates of ED use for potentially preventable kidney disease–related conditions were much lower, ACSC-related ED encounters were associated with important clinical and sociodemographic factors, including younger age, social disadvantage, comorbidities such as chronic pain, and high historical ED use, with half resulting in hospital admission. These findings highlight the burden of acute care use for people receiving dialysis, their families, and the health system and opportunities to address contributors to potentially preventable ED use in community-based settings.

Defined as a set of conditions for which hospitalizations could be avoided through effective outpatient management, ACSCs have been used by researchers and decision-makers as factors in ambulatory care quality.^21,22,31,32^ Previous studies using the kidney disease–specific ACSC classification have also shown high rates of all-cause and potentially preventable acute care use among people with chronic kidney disease, with rates increasing in a graded fashion as disease severity increases.^4,13,23,24^ Our study complements and extends earlier findings by focusing on a subset of individuals with end-stage kidney disease receiving dialysis who have frequent ED use and addressing a limitation of using ACSC diagnoses to define preventable acute care use: the underestimation of other individual- and system-level influences.^33^ Other work has identified important associations between ACSC-related hospitalizations and sociodemographic factors, such as being a member of a racial or ethnic minority group, socioeconomic disadvantage, and rural residence.^22,34,35,36,37^ Our study also found an association between material deprivation and potentially preventable ED use among patients receiving dialysis; however, we identified additional patient characteristics (eg, younger age [≤44 years], chronic pain) that may challenge preventative health measures and accessibility of outpatient care.^38,39,40,41,42,43^ Despite the relatively low rates of ACSC-related ED use, our findings point to important upstream targets to both identify and focus attention on individuals for whom community-based interventions could bring about measurable changes on acute care use and potentially costs, patient care experiences, and clinical outcomes.

In one systematic review assessing factors underlying ED use among people receiving maintenance hemodialysis, included studies reported primarily on dialysis parameters and infrequently on psychosocial and system factors (eg, psychiatric conditions, social determinants of health, or financial models).^44^ Only 4 included studies had evaluated the implications of an upstream intervention, such as home telemonitoring, for ED use.^44^ A separate scoping review further highlights a gap in available acute care avoidance interventions targeted to people experiencing complications of advanced kidney disease, such as hyperkalemia or volume overload.^45^ As these complications are among potentially preventable kidney disease–related ACSCs, their prompt detection and treatment outside of the acute care setting present opportunities for mitigating ED use in this population. The unique requirement for dialysis both as life-sustaining therapy and to treat disease-related complications means that any proposed preventative care strategies should necessarily consider the imminence of dialysis need and supports (eg, resources, expertise) for safe, effective management.

While understanding the risk factors of potentially preventable ED use is grounded in a need for targeted identification and intervention, high overall ED use in patients with kidney failure continues to burden health systems internationally. With half of ACSC-related ED encounters resulting in hospital admission, the potential cost implications of our findings are significant, as patients receiving dialysis account for disproportionately high spending on both all-cause and kidney disease–specific ACSC hospitalizations compared with people with lower risk or no kidney disease.^46^ Moreover, one-third of patients in our cohort had more than 3 ED encounters per year, reflecting a high-use subset of patients with more comorbidities, greater socioeconomic disadvantage, and increased likelihood of dying during follow up than low-frequency ED users. Thus, concerted health promotion efforts involving not only dialysis care teams but also community disciplines (eg, primary care, mental health services) are vital for addressing social determinants of health, promoting continuity of care, and managing complexity among those at risk for excess acute care use.^47^

### Limitations

Our study should be interpreted in light of its limitations. First, included covariates were limited to measurable factors captured within our dataset. While we included several factors in high health care use from Andersen’s behavioral framework,^25^ we were unable to assess the contribution of certain predisposing, enabling, and need factors (eg, religion, employment status, or treatment adherence). Second, we did not assess the influence of dialysis modality–specific characteristics (eg, ultrafiltration rate) on ACSC-related or all-cause ED encounters. Third, our cohort included only individuals receiving maintenance dialysis for more than 90 days, and thus we cannot ascertain the influence of potentially preventable conditions around the time of dialysis initiation. Others have reported increased rates of ED use during periods just before or after initiating dialysis related to complications of advanced kidney disease or uremia. Fourth, as kidney disease–specific ACSCs represent a small portion of overall ACSCs defined for general use, their application in this study may underestimate the total burden of potentially preventable acute care use. Lastly, while we were able to crudely assess specialist and primary care follow-up, we cannot confidently point to gaps in continuity of outpatient care or involvement of multidisciplinary team members in addressing the identified medical and sociodemographic factors associated with potentially preventable ED use in the dialysis population.

## Conclusions

The results of this cohort study show that important medical and sociodemographic factors, such as chronic pain and socioeconomic disadvantage, are associated with potentially preventable ED use among people receiving maintenance dialysis. While potentially preventable ED encounters reflected a small proportion of ED visits overall, our findings point to a need for holistic care approaches in this population that improve access to care for dialysis-related and other urgent health concerns and attend to patients’ well-being and psychosocial concerns. Our study raises important questions about the aspects of health care use that are truly preventable and to what extent efforts to curb frequent ED use in this population should target the condition itself, its underlying contributors, or acute care processes.

## Notes

Supplement 1. **eTable 1.** *ICD-9-CM* and *ICD-10-CA* Codes Used to Define the Study Cohort and Covariates **eTable 2.** Diagnosis Most Responsible for the First ED Encounter During the Study Period, Stratified by ACSC Presentation (n=3877 ED Encounters Among 3877 Patients) **eTable 3.** Diagnosis Most Responsible for All ED Encounters During the Study Period, Stratified by ACSC Presentation (n=34 029 ED Encounters Among 3877 Patients) **eTable 4.** Association Between Characteristics and the Rate of ACSC ED Encounters Among Adults Receiving Maintenance Dialysis Between April 1, 2010, and March 31, 2019, Who Had at Least 1 ED Encounter (n=3877) **eTable 5.** Association Between Characteristics and the Rate of ACSC ED Encounters Among Adults Receiving Maintenance Dialysis Between April 1, 2010, and March 31, 2019 (n=4925) **eFigure.** Sensitivity Analysis of Factors Associated With Potentially Preventable ED Encounters Among All Patients Receiving Maintenance Dialysis (n=4925)

Supplement 2.
Data Sharing Statement
