# Supplementary material for: Emergency Department Use Among Adults Receiving Dialysis
Source: JAMA Netw Open. 2024 May 29;7(5):e2413754. doi: 10.1001/jamanetworkopen.2024.13754 (PMC11137633; doi:10.1001/jamanetworkopen.2024.13754)

Supplemental Online Content

Ronksley PE, Scory TD, McRae AD, et al. Emergency department use among adults receiving dialysis in Canada. *JAMA Netw Open*. 2024;7(5):e2413754. doi:10.1001/jamanetworkopen.2024.13754

eTable 1. *ICD-9-CM* and *ICD-10-CA* Codes Used to Define the Study Cohort and Covariates

eTable 2. Diagnosis Most Responsible for the First ED Encounter During the Study Period, Stratified by ACSC Presentation (n = 3,877 ED Encounters Among 3,877 Patients)

eTable 3. Diagnosis Most Responsible for All ED Encounters During the Study Period, Stratified by ACSC Presentation (n = 34,029 ED Encounters Among 3,877 Patients)

eTable 4. Association Between Characteristics and the Rate of ACSC ED Encounters Among Adults Receiving Maintenance Dialysis Between April 1, 2010, and March 31, 2019, Who Had at Least 1 ED Encounter (n = 3,877)

eTable 5. Association Between Characteristics and the Rate of ACSC ED Encounters Among Adults Receiving Maintenance Dialysis Between April 1, 2010, and March 31, 2019 (n = 4,925)

eFigure. Sensitivity Analysis of Factors Associated With Potentially Preventable ED Encounters Among All Patients Receiving Maintenance Dialysis (n = 4,925)

This supplemental material has been provided by the authors to give readers additional information about their work.

eTable 1. *ICD-9-CM* and *ICD-10-CA* Codes Used to Define the Study Cohort and Covariates

Ambulatory care sensitive condition	ICD-9-CM	ICD-10-CA
Heart failure	398.90 398.91 402.01 402.11 402.91 404.01 404.03 404.11 404.13 404.91 404.93 414.8 425.4-425.9 428.0-428.4 428.9	I09.9 I25.5 I42.0 I42.5-I42.9 I43.0-I43.2 I43.8 I50.0 I50.1 I50.9
Hyperkalemia	276.7	E87.5
Volume overload	276.6	E87.7
Malignant hypertension	401.0 402.0 403.0 404.0 405.0	I10.1
**Comorbidity**	**ICD-9-CM**	**ICD-10-CA**
Acute myocardial infarction	410	I21, I22
Atrial fibrillation	427.3	I48.0
Cancer	153-154, 162-163, 174, 180, 185, 230.3-230.6, 231.2, 233.0-233.1, 233.4	C18-C21, C33-C34, C38.4, C45.0, C46.71, C50, C53, C61, D01.0-D01.3, D02.2, D05-D06, D07.5
Chronic pain	307.80, 307.89, 338.0, 338.2, 338.4, 719.41, 719.45 - 719.47, 719.49, 720.0, 720.2, 720.9, 721.0 - 721.4, 721.6, 721.8, 721.9, 722, 723.0, 723.1, 723.3 - 723.9, 724.0 - 724.6, 724.70, 724.79, 724.8, 724.9, 729.0 - 729.2, 729.4, 729.5	F45.4, M08.1, M25.50, M25.51, M25.55 - M25.57, M43.2 - M43.6, M45, M46.1, M46.3, M46.4, M46.9, M47, M48.0, M48.1, M48.8, M48.9, M50.8, M50.9, M51, M53.1 - M53.3, M53.8, M53.9, M54, M60.8, M60.9, M63.3, M79.0 - M79.2, M79.6, M79.7, M96.1
Chronic pulmonary disease	416.8, 416.9, 490–492, 494-505, 506.4, 508.1, 508.8	I27.8, I27.9, J40–J44, J46-J47, J60–J67, J68.4, J70.1, J70.3
Constipation, severe	560.1, 560.30, 560.39, 560.9, 564.0, 569.83, 569.89	K55.8, K56.0, K56.4, K56.7, K59.0, K63.1, K63.4, K63.81, K63.88, K92.80, K92.88
Depression	296.2, 296.3, 296.5, 300.4, 309, 311	F20.4, F31.3–F31.5, F32, F33, F34.1, F41.2, F43.2
Dementia	290, 294.1, 331.2	F00–F03, F05.1, G30, G31.1
Diabetes mellitus	250	E10-E14
Hypertension	401-405	I10-I13, I15
Hypothyroidism	240.9, 243, 244, 246.1, 246.8	E00–E03, E89.0
Peripheral vascular disease	443.9, 440.2	I73.9, I70.2, I79.2
Stroke	430-434	I60-I64

Note: Comorbidities were defined using an unrestricted lookback period, except cancer (defined using a five-year lookback period).

eTable 2. Diagnosis Most Responsible for the First ED Encounter During the Study Period, Stratified by ACSC Presentation (n = 3,877 ED Encounters Among 3,877 Patients)

Diagnosis	ICD-10-CA Code	n (%)
**Overall (n = 3,877)**		
Chest pain, unspecified	R07.4	106 (2.7)
Abdominal pain, unspecified	R10.4	80 (2.1)
Syncope and collapse [includes blackout, fainting]	R55	70 (1.8)
Pneumonia, unspecified	J18.9	69 (1.8)
Sepsis, unspecified	A41.9	67 (1.7)
Malaise and fatigue [includes asthenia, debility, general physical deterioration, lethargy, tiredness]	R53	64 (1.7)
Gastroenteritis and colitis of unspecified origin	A09.9	58 (1.5)
Urinary tract infection, site not specified	N39.0	58 (1.5)
Chronic kidney disease, unspecified	N18.9	56 (1.4)
Dyspnea [includes orthopnea, shortness of breath]	R06.0	55 (1.4)
**Non-ACSC Presentation (n = 3,122)**		
Chest pain, unspecified	R07.4	92 (2.9)
Sepsis, unspecified	A41.9	63 (2.0)
Abdominal pain, unspecified	R10.4	63 (2.0)
Pneumonia, unspecified	J18.9	58 (1.9)
Syncope and collapse [includes blackout, fainting]	R55	58 (1.9)
Malaise and fatigue [includes asthenia, debility, general physical deterioration, lethargy, tiredness]	R53	54 (1.7)
Urinary tract infection, site not specified	N39.0	48 (1.5)
Gastroenteritis and colitis of unspecified origin	A09.9	45 (1.4)
Chronic kidney disease, unspecified	N18.9	45 (1.4)
Chronic kidney disease, stage 5	N18.5	43 (1.4)
**ACSC Presentation (n = 755)**		
Congestive heart failure	I50.0	51 (6.8)
Hyperkalemia	E87.5	42 (5.6)
Fluid overload	E87.7	20 (2.6)
Dyspnea [includes orthopnea, shortness of breath]	R06.0	18 (2.4)
Abdominal pain, unspecified	R10.4	17 (2.3)
Chest pain, unspecified	R07.4	14 (1.9)
Gastroenteritis and colitis of unspecified origin	A09.9	13 (1.7)
Type 2 diabetes mellitus with established or advanced kidney disease	E11.23	12 (1.6)
Syncope and collapse [includes blackout, fainting]	R55	12 (1.6)
Pneumonia, unspecified	J18.9	11 (1.5)

Note: Patients are categorized as having an ACSC presentation if they experienced an ACSC at any time during the follow-up period; the first ED encounter may not necessarily have been an ACSC.
Abbreviations: ACSC, ambulatory care sensitive condition; ED, emergency department

eTable 3. Diagnosis Most Responsible for All ED Encounters During the Study Period, Stratified by ACSC Presentation (n = 34,029 ED Encounters Among 3,877 Patients)

Diagnosis	ICD-10-CA Code	n (%)
**Overall (n = 34,029)**		
Chemotherapy [includes antibiotic therapy, maintenance chemotherapy]	Z51.2	2,442 (7.2)
Chest pain, unspecified	R07.4	799 (2.3)
Abdominal pain, unspecified	R10.4	723 (2.1)
Pneumonia, unspecified	J18.9	627 (1.8)
Hyperkalemia	E87.5	572 (1.7)
Malaise and fatigue [includes asthenia, debility, general physical deterioration, lethargy, tiredness]	R53	552 (1.6)
Attention to surgical dressings and sutures [includes change of dressings, removal of sutures]	Z48.0	506 (1.5)
Congestive heart failure	I50.0	497 (1.5)
Sepsis, unspecified	A41.9	471 (1.4)
Abnormalities of breathing, dyspnoea	R06.0	455 (1.3)
**Non-ACSC Presentation (n = 21,035)**		
Chemotherapy [includes antibiotic therapy, maintenance chemotherapy]	Z51.2	1,681 (8.0)
Chest pain, unspecified	R07.4	475 (2.3)
Abdominal pain, unspecified	R10.4	467 (2.2)
Pneumonia, unspecified	J18.9	374 (1.8)
Malaise and fatigue [includes asthenia, debility, general physical deterioration, lethargy, tiredness]	R53	358 (1.7)
Attention to surgical dressings and sutures [includes change of dressings, removal of sutures]	Z48.0	350 (1.7)
Sepsis, unspecified	A41.9	343 (1.6)
Urinary tract infection, site not specified	N39.0	312 (1.5)
Gastroenteritis and colitis of unspecified origin	A09.9	267 (1.3)
Benign hypertension	I10.0	242 (1.2)
**ACSC Presentation (n = 12,994)**		
Chemotherapy [includes antibiotic therapy, maintenance chemotherapy]	Z51.2	761 (5.9)
Hyperkalemia	E87.5	572 (4.4)
Congestive heart failure	I50.0	497 (3.8)
Chest pain, unspecified	R07.4	324 (2.5)
Abdominal pain, unspecified	R10.4	256 (2.0)
Pneumonia, unspecified	J18.9	253 (1.9)
Dyspnea [includes orthopnea, shortness of breath]	R06.0	235 (1.8)
Fluid overload	E87.7	231 (1.8)
Chronic kidney disease, stage 5	N18.5	211 (1.6)
Malaise and fatigue [includes asthenia, debility, general physical deterioration, lethargy, tiredness]	R53	195 (1.5)

Note: Patients are categorized as having an ACSC presentation if they experienced an ACSC at any time during the follow-up period; the first ED encounter may not necessarily have been an ACSC.
Abbreviations: ACSC, ambulatory care sensitive condition; ED, emergency department

eTable 4. Association Between Characteristics and the Rate of ACSC ED Encounters Among Adults Receiving Maintenance Dialysis Between April 1, 2010, and March 31, 2019, Who Had at Least 1 ED Encounter (n = 3,877)

Predictor variable	Number of Events	Total Person-Years	Crude IRR (95% CI)	Adjusted IRR (95% CI)
Age, categorical				
18 to < 45	325	1,806.1	1.52 (1.21-1.92)	1.70 (1.33-2.15)
45 to < 65	542	4,740.6	1.10 (0.92-1.33)	1.11 (0.92-1.33)
≥ 65	484	4,400.4	Reference	Reference
Sex				
Male	795	6,619.2	0.98 (0.83-1.16)	
Female	556	4,327.9	Reference	
Residence				
Rural	338	2,062.5	1.30 (1.06-1.59)	
Urban	1010	8,866.8	Reference	
Missing	3	17.8	1.26 (0.20-7.66)	
Income quintile, before tax				
1 (Lowest quintile)	551	3,654.3	1.61 (1.19-2.17)	
2	340	2,635.9	1.35 (0.99-1.85)	
3	193	1,842.8	1.10 (0.78-1.54)	
4	152	1,601.1	1.06 (0.74-1.50)	
5 (Highest quintile)	112	1,190.2	Reference	
Missing	3	22.8	1.31 (0.22-7.68)	
Pampalon material deprivation index				
1 (Least deprived)	105	1,165.8	Reference	Reference
2	186	1,529.9	1.28 (0.90-1.82)	1.24 (0.87-1.75)
3	221	1,897.0	1.32 (0.94-1.86)	1.28 (0.91-1.78)
4	180	2,195.8	0.85 (0.60-1.20)	0.86 (0.61-1.20)
5 (Most deprived)	557	3,395.3	1.70 (1.25-2.31)	1.57 (1.16-2.12)
Missing	102	763.3	1.52 (1.01-2.29)	1.30 (0.87-1.95)
Pampalon social deprivation index				
1 (Least deprived)	208	1,510.1	Reference	
2	140	1,362.0	0.79 (0.57-1.10)	
3	215	1,858.7	0.78 (0.58-1.06)	
4	286	2,376.2	0.85 (0.64-1.13)	
5 (Most deprived)	400	3,076.7	0.96 (0.73-1.25)	
Missing	102	763.3	1.03 (0.71-1.51)	
Initial dialysis modality				
Hemodialysis	951	6,993.1	1.38 (1.16-1.65)	
Peritoneal dialysis	400	3,953.9	Reference	
Modality switch				
Yes	348	3,215.6	0.82 (0.68-0.99)	
No	1003	7,731.4	Reference	
Dialysis vintage				
≥ 12 months	1,259	10,480.8	0.61 (0.47-0.80)	
< 12 months	92	466.3	Reference	
Acute myocardial infarction	187	1,186.0	1.38 (1.07-1.79)	

Predictor variable	Number of Events	Total Person-Years	Crude IRR (95% CI)	Adjusted IRR (95% CI)
Atrial fibrillation	194	1,502.7	1.10 (0.87-1.39)	
Cancer	57	791.2	0.57 (0.40-0.81)	0.59 (0.42-0.85)
Chronic pain	852	5,983.3	1.47 (1.24-1.74)	1.35 (1.14-1.61)
Chronic pulmonary disease	471	3,012.9	1.41 (1.18-1.69)	
Constipation, severe	217	1,248.8	1.46 (1.15-1.86)	
Dementia	55	458.3	0.97 (0.65-1.46)	
Depression	527	3,382.4	1.42 (1.20-1.69)	
Diabetes mellitus	936	6,743.0	1.43 (1.20-1.71)	
Heart failure	631	4,188.2	1.52 (1.28-1.79)	1.50 (1.26-1.79)
Hypertension	1249	10,003.6	1.17 (0.86-1.60)	
Hypothyroidism	198	1,570.3	0.97 (0.77-1.23)	
Peripheral vascular disease	170	1,298.9	1.04 (0.81-1.34)	
Stroke	278	2,352.3	0.99 (0.80-1.21)	
Polypharmacy[a]				
Yes	1127	8,536.2	1.37 (1.11-1.69)	
No	224	2,410.8	Reference	
Potassium level				
Low	73	839.7	0.68 (0.48-0.96)	0.67 (0.48-0.95)
Normal	833	7,281.8	Reference	Reference
High	438	2,752.2	1.36 (1.13-1.64)	1.31 (1.09-1.58)
No test	7	73.4	0.82 (0.26-2.55)	0.93 (0.30-2.87)
Sodium level				
Low	513	4,261.0	1.01 (0.85-1.20)	
Normal	809	6,506.4	Reference	
High	25	114.0	1.53 (0.72-3.28)	
No test	4	65.7	0.46 (0.12-1.74)	
A1C level				
Normal	321	2,672.5	Reference	
Pre-diabetic	117	910.7	1.00 (0.72-1.40)	
Diabetic	592	3,983.1	1.18 (0.95-1.46)	
No test	321	3,380.7	0.76 (0.60-0.96)	
Number of general practitioner visits				
≥ 1	1262	10,213.7	0.96 (0.69-1.35)	
0	89	733.3	Reference	
Primary care attachment in prior year				
Low (0 – 50%)	138	838.9	1.35 (0.99-1.83)	
Medium (50 – 75%)	316	2,436.7	1.08 (0.88-1.34)	
High (75 – 100%)	628	5,334.9	Reference	
Missing	269	2,336.6	0.99 (0.79-1.23)	
Number of cardiologist visits				
≥ 1	409	3,141.6	1.08 (0.90-1.30)	
0	942	7,805.5	Reference	
Number of ED visits				
≥ 3	779	4,739.6	2.66 (2.04-3.46)	1.46 (1.23-1.73)
< 3	572	6,207.5	Reference	Reference

Predictor variable	Number of Events	Total Person-Years	Crude IRR (95% CI)	Adjusted IRR (95% CI)
Number of hospitalizations in prior year				
≥ 1	1086	7,715.6	1.72 (1.41-2.09)	
0	265	3,231.5	Reference	
Cumulative hospital LOS in prior year				
≥ 14 days	615	4,252.5	1.14 (0.94-1.38)	
< 14 days	473	3,488.8	Reference	
No hospitalization	263	3,205.8	0.63 (0.50-0.78)	
Prior ICU hospitalization				
Yes	303	1,960.4	1.37 (1.11-1.69)	
No	1048	8,986.7	Reference	
LTC placement in prior year				
Yes	40	381.7	0.86 (0.55-1.35)	
No	1311	10,565.4	Reference	

Note: Crude rate ratios were estimated using univariable negative binomial regression models. All statistically significant predictors at α = 0.05 were included in the initial adjusted model. Predictors that were not statistically significant (also at α = 0.05) in the full adjusted model were removed. Then, likelihood ratio tests and AIC values were used to determine the goodness of fit, and the best model is presented.

Abbreviations: CI, confidence interval; ED, emergency department; ICU, intensive care unit; LOS, length of stay; LTC, long-term care; PY, person-years; IRR, incidence rate ratio; SD, standard deviation.

[a]Defined as receipt of 5 or more unique prescription medications within Alberta's Pharmaceutical Information Network data set.

eTable 5. Association Between Characteristics and the Rate of ACSC ED Encounters Among Adults Receiving Maintenance Dialysis Between April 1, 2010, and March 31, 2019 (n = 4,925)

Predictor variable	Number of Events	Total Person-Years	Crude IRR (95% CI)	Adjusted IRR (95% CI)
Age, categorical				
18 to < 45	325	2,013.1	1.46 (1.16-1.83)	1.74 (1.37-2.21)
45 to < 65	542	5,278.5	1.08 (0.89-1.29)	1.10 (0.92-1.33)
≥ 65	484	4,810.3	Reference	Reference
Sex				
Male	795	7,397.1	0.95 (0.80-1.12)	
Female	556	4,704.8	Reference	
Residence				
Rural	338	2,206.5	1.36 (1.11-1.66)	
Urban	1010	9,875.3	Reference	
Missing	3	20.1	1.19 (0.21-6.65)	
Income quintile, before tax				
1 (Lowest quintile)	551	4,014.2	Reference	
2	340	2,882.4	0.84 (0.68-1.04)	
3	193	2,048.7	0.67 (0.52-0.86)	
4	152	1,756.8	0.66 (0.51-0.86)	
5 (Highest quintile)	112	1,374.6	0.59 (0.44-0.79)	
Missing	3	25.1	0.78 (0.14-4.24)	
Pampalon material deprivation index				
1 (Least deprived)	105	1,330.2	Reference	Reference
2	186	1,716.0	1.30 (0.92-1.84)	1.23 (0.87-1.74)
3	221	2,096.0	1.38 (0.99-1.93)	1.25 (0.89-1.74)
4	180	2,409.6	0.90 (0.64-1.26)	0.84 (0.60-1.18)
5 (Most deprived)	557	3,723.9	1.78 (1.32-2.41)	1.53 (1.13-2.07)
Missing	102	826.3	1.64 (1.09-2.45)	1.30 (0.87-1.95)
Pampalon social deprivation index				
1 (Least deprived)	208	1,703.9	Reference	
2	140	1,498.4	0.82 (0.59-1.14)	
3	215	2,058.0	0.81 (0.60-1.09)	
4	286	2,601.2	0.89 (0.67-1.18)	
5 (Most deprived)	400	3,414.0	0.99 (0.76-1.28)	
Missing	102	826.3	1.11 (0.76-1.61)	
Initial dialysis modality				
Hemodialysis	951	7,709.4	1.39 (1.17-1.65)	
Peritoneal dialysis	400	4,392.5	Reference	
Modality switch				
Yes	348	3,457.5	0.87 (0.72-1.05)	
No	1003	8,644.4	Reference	
Dialysis vintage				
≥ 12 months	1259	11,401.0	0.86 (0.67-1.10)	
< 12 months	92	700.9	Reference	
Acute myocardial infarction	187	1,259.1	1.45 (1.12-1.87)	

Predictor variable	Number of Events	Total Person-Years	Crude IRR (95% CI)	Adjusted IRR (95% CI)
Atrial fibrillation	194	1,598.0	1.16 (0.92-1.47)	
Cancer	57	864.2	0.58 (0.41-0.83)	0.59 (0.42-0.84)
Chronic pain	852	6,425.7	1.58 (1.34-1.87)	1.35 (1.14-1.60)
Chronic pulmonary disease	471	3,181.1	1.52 (1.28-1.82)	
Constipation, severe	217	1,304.4	1.58 (1.24-2.01)	
Dementia	55	491.9	1.01 (0.67-1.50)	
Depression	527	3,650.2	1.48 (1.25-1.76)	
Diabetes mellitus	936	7,297.9	1.53 (1.29-1.82)	
Heart failure	631	4,470.5	1.62 (1.37-1.92)	1.50 (1.26-1.79)
Hypertension	1249	10,971.8	1.29 (0.95-1.73)	
Hypothyroidism	198	1,679.6	1.01 (0.80-1.28)	
Peripheral vascular disease	170	1,394.9	1.09 (0.85-1.40)	
Stroke	278	2,463.3	1.07 (0.88-1.31)	
Polypharmacy[a]				
Yes	1127	9,349.8	1.43 (1.16-1.76)	
No	224	2,752.1	Reference	
Potassium level				
Low	73	839.7	0.68 (0.48-0.96)	0.67 (0.48-0.94)
Normal	833	7,281.8	Reference	Reference
High	438	2,752.2	1.36 (1.13-1.64)	1.31 (1.09-1.58)
No test	7	1,228.3	0.05 (0.02-0.10)	0.05 (0.03-0.12)
Sodium level				
Low	513	4,261.0	1.01 (0.85-1.20)	
Normal	809	6,506.4	Reference	
High	25	114.0	1.54 (0.72-3.29)	
No test	4	1,220.5	0.02 (0.01-0.07)	
A1C level				
Normal	321	2,672.5	Reference	
Pre-diabetic	117	910.7	1.00 (0.71-1.41)	
Diabetic	592	3,983.1	1.18 (0.95-1.47)	
No test	321	4,535.6	0.53 (0.42-0.66)	
Number of general practitioner visits				
≥ 1	1262	11,222.8	1.07 (0.77-1.47)	
0	89	879.2	Reference	
Primary care attachment in prior year				
Low (0 – 50%)	138	907.6	1.37 (1.01-1.86)	
Medium (50 – 75%)	316	2,630.3	1.11 (0.90-1.38)	
High (75 – 100%)	628	5,877.4	Reference	
Missing	269	2,686.6	0.93 (0.75-1.15)	
Number of cardiologist visits				
≥ 1	409	3,434.1	1.10 (0.92-1.32)	
0	942	8,667.8	Reference	
Number of ED visits				
≥ 3	779	4,962.9	1.97 (1.67-2.32)	1.46 (1.23-1.73)
< 3	572	7,139.0	Reference	Reference

Predictor variable	Number of Events	Total Person-Years	Crude IRR (95% CI)	Adjusted IRR (95% CI)
Number of hospitalizations in prior year				
≥ 1	1086	8,327.1	1.88 (1.56-2.28)	
0	265	3,774.8	Reference	
Cumulative hospital LOS in prior year				
≥ 14 days	615	4,520.5	1.19 (0.98-1.44)	
< 14 days	473	3,851.2	Reference	
No hospitalization	263	3,730.2	0.59 (0.48-0.74)	
Prior ICU hospitalization				
Yes	303	2,120.7	1.40 (1.14-1.72)	
No	1048	9,981.2	Reference	
LTC placement in prior year				
Yes	40	400.8	0.93 (0.59-1.46)	
No	1311	11,701.2	Reference	

Note: Crude rate ratios were estimated using univariable negative binomial regression models. All statistically significant predictors at α = 0.05 were included in the initial adjusted model. Predictors that were not statistically significant (also at α = 0.05) in the full adjusted model were removed. Then, likelihood ratio tests and AIC values were used to determine the goodness of fit, and the best model is presented.
Abbreviations: CI, confidence interval; ED, emergency department; ICU, intensive care unit; LOS, length of stay; LTC, long-term care; PY, person-years; IRR, incidence rate ratio; SD, standard deviation.
[a]Defined as receipt of 5 or more unique prescription medications within Alberta's Pharmaceutical Information Network data set

eFigure. Sensitivity Analysis of Factors Associated With Potentially Preventable ED Encounters Among All Patients Receiving Maintenance Dialysis (n = 4,925)

Predictor	No. of Events	Total Person-Years	Adjusted Incidence Rate Ratio (95% CI)
**Age Group**			
18-<45 years	325	2,013	1.74 (1.37-2.21)
45-<65 years	542	5,278	1.10 (0.92-1.33)
65 years and older	484	4,810	Reference
**Pampalon material deprivation index**			
1 (Least deprived)	105	1,330	Reference
2	186	1,716	1.23 (0.87-1.74)
3	221	2,096	1.25 (0.89-1.74)
4	180	2,410	0.84 (0.60-1.18)
5 (Most deprived)	557	3,724	1.53 (1.13-2.07)
Missing	102	826	1.30 (0.87-1.95)
**Chronic pain**			
Yes	852	6,426	1.35 (1.14-1.60)
No	499	5,676	Reference
**Cancer**			
Yes	57	864	0.59 (0.42-0.84)
No	1294	11,238	Reference
**Heart failure**			
Yes	631	4,470	1.50 (1.26-1.79)
No	720	7,631	Reference
**Potassium level**			
Low	73	840	0.67 (0.48-0.94)
Normal	833	7,282	Reference
High	438	2,752	1.31 (1.09-1.58)
No test	7	1,228	0.05 (0.03-0.12)
**Number of ER visits**			
3 or more visits	779	4,963	1.46 (1.23-1.73)
0-2 visits	572	7,139	Reference

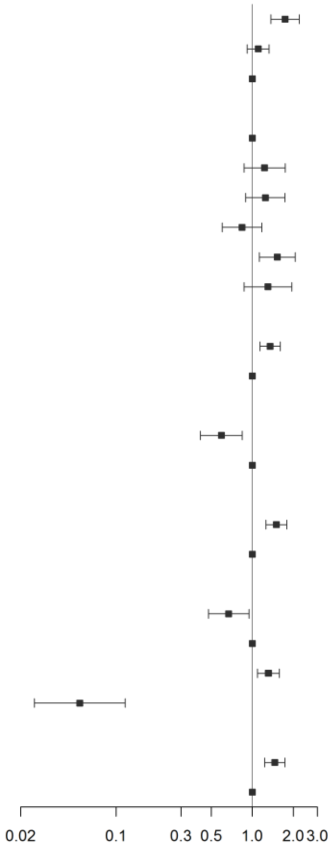

Supplement: Supplement 1. — eTable 1. ICD-9-CM and ICD-10-CA Codes Used to Define the Study Cohort and Covariates eTable 2. Diagnosis Most Responsible for the First ED Encounter During the Study Period, Stratified by ACSC Presentation (n=3877 ED Encounters Among 3877 Patients) eTable 3. Diagnosis Most Responsible for All ED Encounters During the Study Period, Stratified by ACSC Presentation (n=34 029 ED Encounters Among 3877 Patients) eTable 4. Association Between Characteristics and the Rate of ACSC ED Encounters Among Adults Receiving Maintenance Dialysis Between April 1, 2010, and March 31, 2019, Who Had at Least 1 ED Encounter (n=3877) eTable 5. Association Between Characteristics and the Rate of ACSC ED Encounters Among Adults Receiving Maintenance Dialysis Between April 1, 2010, and March 31, 2019 (n=4925) eFigure. Sensitivity Analysis of Factors Associated With Potentially Preventable ED Encounters Among All Patients Receiving Maintenance Dialysis (n=4925) [file jamanetwopen-e2413754-s001.pdf]
