# Supplementary material for: Emergency Department Use Among Adults Receiving Dialysis
Source: JAMA Netw Open. 2024 May 29;7(5):e2413754. doi: 10.1001/jamanetworkopen.2024.13754 (PMC11137633; doi:10.1001/jamanetworkopen.2024.13754)
Supplement: Supplement 2. — Data Sharing Statement [file jamanetwopen-e2413754-s002.pdf]

Data Sharing Statement

Ronksley. Emergency Department Use Among Adults Receiving Dialysis. *JAMA Netw Open*. Published May 29, 2024. doi:10.1001/jamanetworkopen.2024.13754

Data
Data available: No

Additional Information
Explanation for why data not available: We are not able to make our data set available to other researchers due to our contractual arrangements with the provincial health ministry (Alberta Health), who is the data custodian.
